# Effect of silver nanoparticles on the physicochemical and antimicrobial properties of an orthodontic adhesive

**DOI:** 10.1590/1678-775720160154

**Published:** 2016

**Authors:** Felipe Weidenbach DEGRAZIA, Vicente Castelo Branco LEITUNE, Isadora Martini GARCIA, Rodrigo Alex ARTHUR, Susana Maria Werner SAMUEL, Fabrício Mezzomo COLLARES

**Affiliations:** 1 - Universidade Federal do Rio Grande do Sul, Faculdade de Odontologia, Laboratório de Materiais Odontológicos, Porto Alegre, RS, Brasil.; 2 - Universidade Federal do Rio Grande do Sul, Faculdade de Odontologia, Laboratório de Bioquímica e Microbiologia Oral, Porto Alegre, RS, Brasil.

**Keywords:** Metal nanoparticles, Anti-bacterial agents, Shear strength, Dental cements

## Abstract

Orthodontic treatment with fixed brackets plays a major role on the formation of white spot lesions.

**Objective:**

This study aimed to incorporate silver nanoparticle solutions (AgNP) in an orthodontic adhesive and evaluate its physicochemical and antimicrobial properties.

**Material and Methods:**

Silver nanoparticle solutions were added to a commercial adhesive in different concentrations (w/w): 0%, 0.11%, 0.18%, and 0.33%. Shear bond strength (SBS) test was performed after bonding metal brackets to enamel. Raman spectroscopy was used to analyze *in situ* the degree of conversion (DC) of the adhesive layer. The surface free energy (SFE) was evaluated after the measurement of contact angles. Growth inhibition of *Streptococcus mutans* in liquid and solid media was determined by colony-forming unit count and inhibition halo, respectively. One-way ANOVA was performed for SBS, DC, SFE, and growth inhibition.

**Results:**

The incorporation of AgNP solution decreased the SBS (p<0.001) and DC in situ (p<0.001) values. SFE decreased after addition of 0.18% and 0.33% AgNP. Growth inhibition of S. mutans in liquid media was obtained after silver addition (p<0.05).

**Conclusions:**

The addition of AgNP solutions to Transbond™ XT adhesive primer inhibited *S. mutans* growth. SBS, DC, and SFE values decreased after incorporation up to 0.33% AgNP solution without compromising the chemical and physical properties of the adhesive.

## INTRODUCTION

The formation of white spot lesions (WSLs) on dental enamel during orthodontic treatment is considered one of the worst problems faced in dental clinical care. This process mainly occurs due to low oral pH and lactic acid produced by *Streptococcus mutans* metabolism^22^. Previous studies reported an increase of bacterial growth at the interface between adhesive resins used to bond orthodontic attachments to enamel^23,29^. Furthermore, even with patient compliance, the mechanical removal of plaque around orthodontic brackets is difficult and causes an increase of the cariogenic challenge.

The increased appearance of bacteria resistant to commercially antimicrobial agents has led to a crescent necessity for natural and nontoxic sources^16^. Hence, fluoride-based therapy protocols showed moderate evidence of prevention in early tooth decay during fixed brace treatment^9^. Fluoride-releasing materials showed fast decrease of antibacterial activity^24^. In this regard, novel materials aiming to reduce the adhesion of cariogenic streptococci to orthodontic adhesives for longer periods are essential to prevent enamel demineralization.

Silver nanoparticles have been stated to produce higher antimicrobial effect against *S. mutans* with lower concentrations than other agents, such as gold or zinc^17^. This allows for important clinical effects with reduced toxicity. Furthermore, according to Zhang, et al.^30^ (2013), the incorporation of AgNP to an adhesive did not affect the cytotoxicity regarding human gingival fibroblast viability. As shown elsewhere^20^, the antibacterial activity of silver nanoparticle incorporated into adhesive cement may be prolonged up to 4 months. Nevertheless, AgNP powder is difficult to incorporate and disperse into an adhesive. Non-dispersing nanofillers could lead to formation of voids and thus weaken the polymeric matrix. For this reason, a simple method to promote AgNP dispersion with an aqueous solution was recently reported^10^.

Although the addition of water into the non-polymerized resin may harm cross-linking formation, it facilitates silver nanoparticles dispersion and, consequently, improves the antibacterial effect. Previous studies showed growth inhibition effects against *S. mutans* by silver ions release^20^ and bacterial adhesion assay^6^. However, ion release may lead to polymer degradation, whereas the bacterial adhesion assay on adhesive surface does not consider the bacterial colonization that occurs at the interface between enamel and adhesive. Furthermore, studies^1,10^ evaluating mechanical properties of orthodontic adhesives showed no antibacterial assay after the incorporation of low concentrations of silver nanoparticles.

Therefore, the aim of this study was to evaluate the antibacterial effect on liquid and solid media, and physical-chemical properties of an orthodontic adhesive system after incorporation of different concentrations of silver nanoparticle solutions. The null hypothesis was that AgNP incorporation would promote antibacterial activity without compromising the physical-chemical properties of the orthodontic bonding system.

## MATERIAL AND METHODS

### Preparation of experimental adhesives

**Figure 1:**
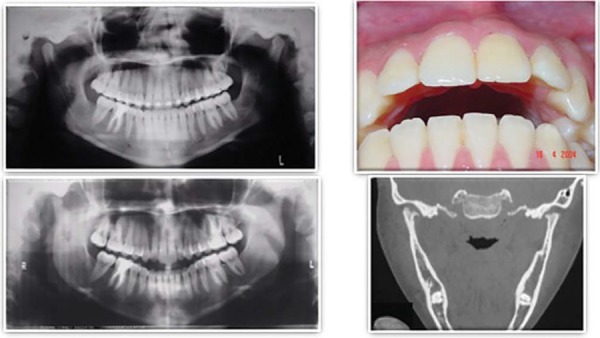
Oval and rhomboid shapes of silver nanoparticles measuring 25-100 nm

To produce the experimental adhesive base, Transbond™ XT primer and adhesive were purchased from 3M Unitek Corp. (Monrovia, CA, USA – LOT N546755 and N556918). Silver nanopowder (particle size <150 nm – Figure 1) was purchased from Sigma-Aldrich Corp. (St. Louis, MO, USA – LOT #MKBP7829V). Bovine mandibular incisors were extracted and stored in distilled water at 4°C for 2 months. Orthodontic metallic brackets for upper central incisors (11.16 mm^2^) were purchased from Dental Morelli Ltd. (Sorocaba, SP, Brazil – LOT 1958344). Attacktec 37% orthophosphoric acid gel was purchased from CaiTHEC Industrial Ltd. (Rio do Sul, SC, Brazil), and acrylic resin was purchased from VIPI Ltd. (Pirassununga, SP, Brazil).

### Preparation of stock solution and dilutions

A stock solution of 11 wt% silver nanoparticles (AgNP) was prepared and mixed with distilled water to facilitate dispersion. The solution was ultrasonicated for 10 min to enhance particle dispersion. The AgNP solution was diluted in Transbond™ XT primer with a Labmate Soft pipette 0.5-10 µL from PZ HTL S/A (Warsaw, MA, Poland). Concentrations of AgNP occurred following a previous study^10^:

0.11% (w/w) – 1:100 dilution (1.0 µL of 11 wt% AgNP solution and 99 µL primer);

0.18% (w/w) – 1.8:100 dilution (1.8 µL of 11 wt% AgNP solution and 98.2 µL primer);

0.33% (w/w) – 3:100 dilution (3.0 µL of 11 wt% AgNP solution and 97 µL primer).

### Shear bond strength (SBS) and adhesive remnant index (ARI)

Forty-eight bovine incisors, free of cracks and caries, were obtained at a slaughterhouse and frozen for a maximum period of 1 month. The labial surface of the bovine incisors crowns (n=12) were polished with #600 and #1200 grid silicon carbide papers for 30 s. Each crown was embedded in self-cured acrylic resin with its long axis perpendicular to the horizontal plane. The embedded crowns were numbered and randomized for each group by the Research Randomizer Form program. The enamel surfaces were etched with acid gel and rinsed with water for 30 s. A thin film of Transbond™ XT primer was applied according to the following groups: control group, with no AgNP solution; 0.11% AgNP; 0.18% AgNP; and 0.33% AgNP. Then, Transbond XT adhesive was applied to the bracket base and the resin was pressed onto the enamel surface. The excess of adhesive was removed with an explorer and light activated for 40 s (10 s for each face) with RadiiCal light emitting diode unit (SDI, Bayswater, VIC, Australia). All bonding procedures were performed according to the manufacturer’s instructions.

After 24 h in distilled water, the teeth were positioned in a Universal Testing Machine Shimadzu EZ-SX (Shimadzu Corp., São Paulo, SP, Brazil). The shear bond strength was tested using a chisel blade with crosshead speed of 0.5 mm/min and 500 N load cell. The ARI score^8^ was analyzed regarding the amount of adhesive on the tooth surface with a stereomicroscope (10x).

### Degree of conversion *in situ* (DC)

**Figure 2:**
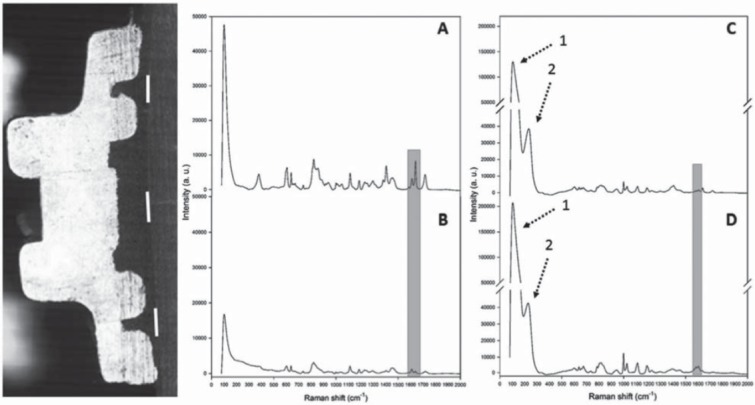
Half of a metal bracket used to analyze degree of conversion (DC) in situ (three lines) before (A) and after (B) polymerization of 0% AgNP; and, before (C) and after (D) polymerization of 0.33% AgNP [highlight of the peaks corresponding to aromatic (1610 cm-1) and aliphatic (1640 cm-1) double bonds]. Arrows show the characteristic Raman peaks of silver compounds: (1) Higher intensity compared with 0% AgNP is due to the Ag lattice vibrational mode (114, 140, 146, and 158 cm-1) and (2) Ag-O stretching/bending modes from AgO2 (230-248 cm-1) and AgCH3COO (258 cm-1)

Orthodontic brackets were bonded to enamel (n=3) as previously described. The specimens remained in distilled water for 24 h and were cut in half by a cutting machine. Each half was positioned on a glass plate fixated by wax. The DC of the primer layer was evaluated at three sites (cervical, medium, and incisal) with micro-Raman spectroscopy (Figure 2) using Senterra equipment (Bruker Optik GmbH, Ettlingen, Baden-Württemberg, Germany). The unpolymerized spectrum of a primer sample was obtained before light activation. The average value of the measurements from each specimen was used to calculate the ratio of double bond content of monomer to polymer shown elsewhere^13^. The characteristic Raman peaks for silver reference compound were previously presented^19^.

### Contact angle (CA) and surface free energy (SFE)

Three specimens of each group were submitted to an optical tensiometer Theta (Biolin Scientific, Stockholm, Sweden) to measure the mean CA (θ) of distilled water (as polar liquid) and of α-bromonaphthalene (as dispersive liquid). The parameters used for water/α-bromonaphthalene drops were: drop out size (2.0 µL), drop rate (2.0 µL/s), and displacement rate of water (20.0 µL/s). The test occurred for 20 s and the Young-Laplace equation was applied to measure θ at the period of 10 s. The SFE calculation (in mN/m) occurred following OWRK/Fowkes* equation with OneAttension software (Biolin Scientific, Stockholm, Sweden).


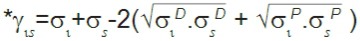


### Growth inhibitory activity in liquid media

Growth inhibition assay occurred in a 96-well plate sterilized by ethylene oxide. Each well was filled with 200 µL of brain-heart infusion (BHI) broth supplemented with 0.5% of sucrose and 20 µL of inoculum, which was prepared after adjusting *Streptococcus mutans* (UA159) to 0.3 density at OD_550nm_. Twelve disk specimens (3.00 mmx2.00 mm) were made (n=3) and placed in contact with inoculum in BHI broth and incubated at 37°C for 24 h. Hence, the disks were transferred to a microtube containing 900 µL of sterile saline solution (0.9% NaCl), and the biofilms were harvested by vortexing. The bacterial suspensions were serially diluted (100 µL) in sterile saline solution. Two aliquots of 25 µL were plated onto BHI agar and incubated for 48 h anaerobically at 37°C, followed by enumeration of the CFU. Statistical analysis was performed by log_10_ CFU/mL.

### Disk diffusion assay

Twelve disks were prepared (3.0 mmx2.00 mm) with the four concentrations of AgNP (n=3). Three disks of each group were placed on agar plates with 150 µL of grown *S. mutans* spread with glass balls. After 48 h of incubation at 37°C, the plates were visually inspected for the presence of inhibitory zones in the bacterial coat. The inhibitory halo of each disk was measured in millimeters.

### Statistical analysis

Statistical analysis was done on Sigma Plot version 12.0 for Windows (Systat Software Inc, San Jose, CA, USA). Normality test was performed with Shapiro-Wilk. One-way ANOVA and Tukey’s *post-hoc* were performed for SBS, DC, SFE, and log_10_CFU/mL. ARI score was evaluated by Kruskal-Wallis. The sample size calculation for each assay was based on previous studies^3,11^.

## RESULTS

Data was normally distributed. The addition of AgNP particles statistically decreased the SBS (p<0.001) and DC *in situ* (p<0.001) values of the commercial adhesive primer when compared with the control group (Table 1).

**Table 1:** Mean and standard deviation of shear bond strength (SBS), degree of conversion (DC) *in situ* and antimicrobial activity against *S. mutans* of different silver concentrations

Groups	SBS*	DC in situ*	Log10CFUΤ
0% AgNP (control)	24.53±4.1^a^	89.50±0.58^a^	7.97±0.17^a^
0.11% AgNP	17.63±3.2^b^	87.51±0.38^b^	5.90±0.66^b^
0.18% AgNP	15.26±2.5^b^	87.44±0.03^b^	5.87±0.59^b^
0.33% AgNP	16.29±2.9^b^	85.92±0.03^c^	5.62±0.71^b^

*Different letters mean statistical difference in the same column (p<0.001)

Τ Different letters mean statistical difference in the same column (p<0.05)

All groups with AgNP addition demonstrated similar antimicrobial activity against *S. mutans*. All groups statistically decreased (p<0.05) *S. mutans* growth compared with control group (Table 1). After 48 h of disk-diffusion assay, no inhibitory halos were detected around disks of any silver concentration.

**Figure 3:**
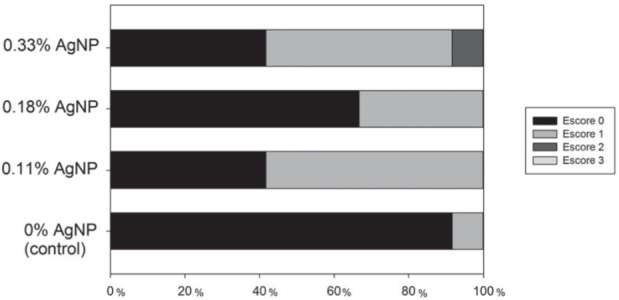
Adhesive remnant index (ARI) score. Decreased amount of adhesive remained on the tooth with 0% AgNP. No statistical difference was found (p>0.05)

Figure 3 shows the ARI scores. The predominant mode of failure was adhesive failure (score 0), which occurred in control group. When AgNP was added to the adhesive primer, higher amount of cohesive failures occurred. No statistical difference was shown (p>0.05).

SFE mean values decreased (p<0.05) compared with control group after addition of 0.18% and 0.33% AgNP (Table 2).

**Table 2:** Mean and standard deviation of contact angle (CA) and surface free energy (SFE) from different concentrations of silver

Groups	Contact Angle		Surface Free Energy
	**Water**	α**-bromo**	
0% AgNP (control)	69.10±5.17	11.16±4.20	50.28±2.24^a^
0.11% AgNP	68.61±4.19	27.73±2.83	48.04±2.55^ab^
0.18% AgNP	74.97±5.56	28.69±4.93	44.33±1.88^b^
0.33% AgNP	75.69±7.00	31.16±4.98	43.05±1.92^b^

Different letters mean statistical difference in the same column (p<0.05)

## DISCUSSION

One of the main reasons for enamel demineralization during orthodontic treatment with fixed appliances is the accumulation of cariogenic biofilm on the enamel/adhesive interface. A large increase in antibiotic-resistant strains and vulnerable aspects of the antimicrobial agents present such as short-term antimicrobial activity and high toxicity, have led researchers to seek new alternative methods. Thus, because of their high reactivity due to their large surface-to-volume ratio, silver nanoparticles play a crucial role in inhibiting bacterial growth in aqueous and solid media^18^. We believe this to be the first time that inhibitory growth effect against *S. mutans* was achieved in liquid media (BHI broth) after incorporation of an aqueous solution with silver nanoparticles into an orthodontic adhesive. The bacterial inhibition growth in liquid media was successfully achieved with all AgNP concentrations.

Recently, a systematic review^5^ demonstrated that the incorporation of antibacterial agents into orthodontic adhesives showed no difference in shear bond strength. One related study^6^ performed antibacterial growth assay in liquid media with 250 and 500 ppm of silver nanoparticles against *S. mutans* without compromising shear bond strength; however, no significant antibacterial growth was found after 24 h. Instead, in our study the inhibitory growth effect of silver nanoparticles was achieved with amounts of 500, 800, and 1500 ppm. The increase concentration of this aqueous solution of AgNP in the adhesive resin was probably the reason for this outcome. The addition of silver nanoparticles in water resulted in a homogenous dispersion through the adhesive. This enhanced distribution improved the antibacterial ability of the composite in spite of decreasing shear bond strength.

The inhibitory effect of silver was previously determined against Gram-positive and Gram-negative cells^21,28^. The DNA molecules become condensed and lose their replication abilities due to a reaction against the denaturation effects of silver ions. Furthermore, silver ions interact with thiol groups in protein, which induce the inactivation of the bacterial proteins^15^. Fan, et al.^14^ (2011) showed inhibition halo against *S. mutans* after incorporation of 0.2 and 0.5% Ag benzoate (AgBz) on a PMMA-based resin blend. Based on the results of our study, the antimicrobial activity of Transbond™ XT primer after incorporating silver nanoparticles was due to direct contact with streptococci without silver ion release. In this study, no inhibition halo was observed around disks after 48 h incubation. The releasing property may harm bond strength longevity and induce adhesive weakness. Furthermore, non-releasing characteristics enable prolongation of the adhesive’s antibacterial effect.

DC *in situ* decreased with higher concentrations of silver nanoparticles. Nonetheless, values of DC in a range between 85 and 90% are related to high cross-linking densities of dental polymers. A previous study^20^ confirmed the similar DC range obtained with its hardness results. The SFE values also decreased after incorporation of 0.18 and 0.33% AgNP. This might have occurred due to lower interaction between the dispersive liquid and the presence of water (polar liquid) in the specimens. The molecular interaction dipole x induced dipole forces between polar liquids and dispersive liquids is known to be weak. Lower SFE values can decrease the interface interaction between the enamel and the adhesive primer, resulting in lower values of SBS. On the other hand, as plaque accumulation around the bracket base has been associated with adhesive rough surface texture^29^, lower values of SFE may prevent bacterial colonization as shown elsewhere^7^.

In accordance to a previous study^25^, water incorporation over 5 vol% to dental adhesives may hinder the formation of an organized polymer network, consequently diminishing its physical properties. In this study, we incorporated an aqueous solution up to 3 vol% and, despite a decrease, the SBS values obtained after incorporation of AgNP solution were greater than the clinically acceptable values between 6-8 MPa^27^. Considering that immediate bond strength to tooth substrate is related to the mechanical properties of the adhesive layer, higher mechanical properties are required to achieve more durable bonding to dental substrate^12^. Hence, the values of SBS shown in this study are consistent with data in the literature^3,26^. The failure pattern shift after incorporation of AgNP may prevent enamel from possible damage. The adhesive failure between enamel and adhesive increases the chance of harming the enamel tissue’s surface.

Despite the fact that the prior application of an adhesive resin has been reported as a step that could be set aside during metal bracket bonding^4^, a particular indication of adhesive resin should be used as an antimicrobial promoter to protect enamel against bacteria. The incorporation of antimicrobial agents into an adhesive resin may be considered a more suitable approach, since it comes into direct contact with the enamel surface^1^. A recent study showed higher amounts of microleakage at the adhesive-enamel interface in different adhesive types^2^. Its lower viscosity and wettability compared with orthodontic composites promote higher dispersion and penetration of antimicrobial agents into the enamel surface.

## CONCLUSION

The incorporation of AgNP solutions into Transbond™ XT adhesive primer showed inhibition growth against *S. mutans*.

Shear bond strength decreased after incorporation of AgNP solution up to 0.33% without compromising the chemical and physical properties of the adhesive primer.
